# Methacrylated Chitosan Methacrylated Poly(vinyl alcohol)-Based Hydrogel Patch for Long-Term Electrochemical Wound pH Sensing

**DOI:** 10.1021/acssensors.4c02172

**Published:** 2025-04-30

**Authors:** Maide Miray Albay, Taher Abbasiasl, ¸Çiğdem Buse Oral, Levent Beker

**Affiliations:** † Department of Biomedical Sciences and Engineering, 52979 Koç University, Rumelifeneri Yolu, Sarıyer, Istanbul 34450, Turkey; ‡ Department of Mechanical Engineering, Koç University Research Center for Translational Research (KUTTAM), Koç University Nanofabrication and Nanocharacterization Center for Scientific and Technological Advanced Research (n2Star), Koç University, Rumelifeneri Yolu, Sarıyer, Istanbul 34450, Turkey

**Keywords:** wound monitoring, hydrogel, capillary, biodegradable, pH, methacrylation

## Abstract

While every wound has the potential to become chronic, the risk is significantly higher in individuals with specific medical conditions. Given this inherent risk, continuous wound monitoring patches are beneficial for all wound types throughout the healing process, enabling the early detection and management of chronic wound development. In this work, we introduce an eco-friendly, hydrogel-integrated, capillary-driven wound patch designed for continuous pH monitoring. The hydrogel, synthesized from methacrylated chitosan and methacrylated poly­(vinyl alcohol), provides antibacterial properties, tissue adhesion, and moisture retention, thereby supporting stable electrochemical pH detection. A paper-based, capillary-driven microfluidic layer facilitates fluid transport toward an evaporation pad, enhancing liquid uptake by approximately 4-fold after 2 h compared to the hydrogel alone. In vitro experiments demonstrated that the hydrogel-integrated sensor effectively monitored pH, exhibiting a near-linear voltage response of 16.92 mV per pH unit. The implementation of such a wound dressing represents a significant advancement in wound healing applications, combining functionality with environmental sustainability.

## Graphical abstract

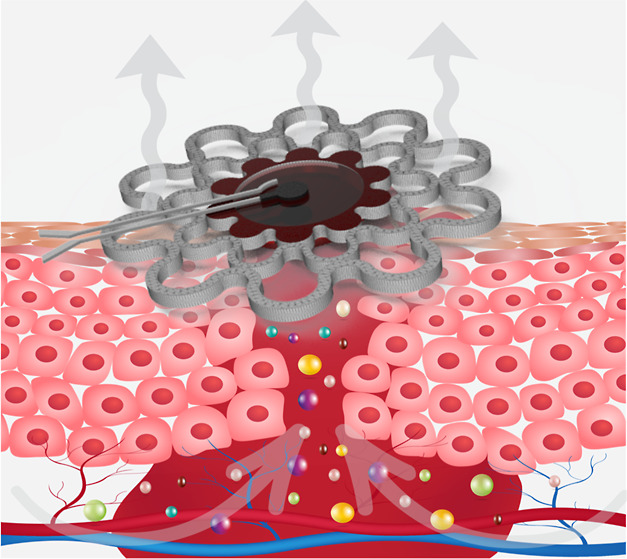

Skin, the largest organ in the human body, is naturally vulnerable to a wide range of external variables. It acts as a protective barrier, defending the body against external factors, such as pathogens, physical trauma, heat, and UV radiation, making it susceptible to numerous wound types. Acute wounds typically follow systematic and timely phases that lead to anatomical and functional restoration, thereby obviating the need for clinical care. In response to a typical acute wound, the mammalian body undergoes three overlapping stages: inflammation, new tissue development, and remodeling.^1^ Unlike acute wounds, chronic wounds do not progress through these stages, resulting in incomplete healing.^2^ Chronic wounds are classified as vascular ulcers (e.g., venous and arterial ulcers), diabetic ulcers, and pressure ulcers.^3^ These types of wounds pose a huge financial burden on healthcare systems, particularly in societies with increasing elderly populations.^4^ According to a 2017 report, the financial cost of managing chronic wounds in the United States is approximately $20 billion annually.^5^ Moreover, in the United States, the prevalence of chronic wounds is notably high, affecting an estimated 2.4 to 4.5 million individuals in 2015,^6^ highlighting the need for improved wound infection monitoring and prevention strategies to reduce these significant clinical and economic burdens.

Failure to appropriately treat any injury can lead to chronicity, underscoring the need for effective wound care to prevent chronic conditions and facilitate the healing process.^7^ Thus, the enhancement of wound healing necessitates the utilization of wound dressing materials that possess many crucial characteristics, including the ability to adhere to the skin, exhibit mechanical stability, retain moisture, absorb exudate, possess antibacterial qualities, and be cost-effective.^8^ Traditional wound dressing materials, such as wool and gauze, are widely used owing to their low cost and ease of manufacturing. However, they do not meet the aforementioned parameters, prompting a significant shift toward tailored wound materials due to their promising features. Chitosan, a biodegradable polymer, is regarded as an appealing choice for wound healing applications because of its naturally antibacterial qualities.^9^ In addition to its polymeric advantages, chitosan hydrogel can facilitate wound hydration and provide a moist environment to improve the healing process. Moreover, chitosan-based hydrogels can facilitate gaseous exchange, maintain an optimal moisture level, and absorb wound exudate.^10,11^ There are already commercially available chitosan-based wound healing technologies such as ChitoFlex by HemCon Medical Technologies,^12^ Tegasorb by 3M,^13^ and Chitoseal by Abbott.^9^ However, these products primarily focus on healing and lack wound monitoring capabilities.

Leveraging continuous wound monitoring can enhance the quality of wound treatment by providing insights into the state of the wound through tracking dynamic changes in wound exudate, including variations in glucose,^14^ uric acid,^15^ temperature,^16^ and enzymatic biomarkers.^17^ Healing stages involve several chemical interactions, which cause pH changes based on the healing phase.^18^ Unlike a typical wound healing process, in which the pH rises to 8 during the granulation phase and then falls to 4–6, an abnormal healing process causes the pH to swing between 7 and 8.^19^ Thus, monitoring pH levels can serve as a valuable indicator of the chronic wound status and the various stages of healing, aiding in optimal wound care. However, during the granulation phase, the wound naturally ceases to produce exudate as the earlier stages provide the most informative characteristics.^20^ Electrochemical sensors leverage the recent advancements in flexible electronics and biomedical devices to offer real-time and noninvasive monitoring of wound pH levels, enabling the fabrication of low-cost, affordable, easy-to-use, and sensitive wound care platforms.^21–24^ However, they face challenges such as degradation and electrode poisoning from proteins and chemokines in wound exudate, which can decrease their sensitivity and accuracy^25^ and cause inflammation over time.^26^ On the other hand, establishing a proper sensor-to-skin contact could be a challenging task due to the nonuniform structure of the skin. Integrating hydrogels with sensors enables the development of a skin-conformal patch, which improves attachment to the skin surface.^27^ Additionally, hydrogels can enhance biomarker detection by increasing the signal quality by maintaining the wound fluid on the sensor area due to their high water-absorption capacity.^28^ Furthermore, hydrogels’ advanced properties, such as porous structure for gaseous exchange,^29^ antimicrobial activity,^30^ improved hemostatic sealant,^31^ and anti-inflammatory and antioxidant characteristics,^32^ make them desirable for wound healing applications. Moreover, they can be used to monitor wound biomarkers such as pH by incorporating dyes into hydrogel matrices.^25,33,34^

Herein, we develop a biodegradable hydrogel-integrated wearable patch designed for continuous electrochemical sensing of wound pH. The hydrogel layer allows continuous liquid absorption, enabling proper contact between the electrochemical sensors and the wound exudate. The patch incorporates chitosan in its hydrogel composition due to its notable antibacterial properties. To enhance the hydrogel’s mechanical strength and adhesiveness, poly­(vinyl alcohol) (PVA) is combined with chitosan, ensuring robust and long-lasting attachment to the skin and sensor. To the best of our knowledge, the combination of methacrylated chitosan and methacrylated PVA has not been reported. Polyhydroxybutyrate/polyhydroxyvalerate (PHB/PHV) is selected as the substrate layer because of its superior biodegradability and flexibility, making it ideal for wound dressing applications. A layer of cellulose paper is integrated into the patch, which enables liquid collection from the hydrogel, followed by evaporation, thus delaying saturation and facilitating long-term continuous monitoring. Moreover, the dimensions of each component in the patch can be tailored by altering the size of the hydrogel, evaporation pad, and sensor to adapt to different wound shapes and sizes. To realize a cost-effective and disposable patch for wound care, the pH sensor is fabricated through screen-printing technology. Our in vitro experiments demonstrate that the wearable patch, which incorporates paper and hydrogel, performs effectively in detecting pH levels by electrochemical sensing. This patch has the potential to be a valuable tool for managing wound care as it can continuously monitor pH levels.

## Results and Discussion

The components of the wound patch and their integration are shown in Figure 1a. To demonstrate the effectiveness of this system, we first synthesized a hydrogel using chitosan and PVA, methacrylating them to avoid using toxic chemicals for cross-linking. Methacrylated chitosan (Meth-Chi) and methacrylated PVA (Meth-PVA) were synthesized separately and then cross-linked by mixing them in a 1:1 ratio and exposing the mixture to UV irradiation (Figure 1b). After synthesizing Meth-Chi/Meth-PVA, it was integrated with pH sensors for wound exudate absorption, followed by integration with the evaporation pad.

**Figure 1:**
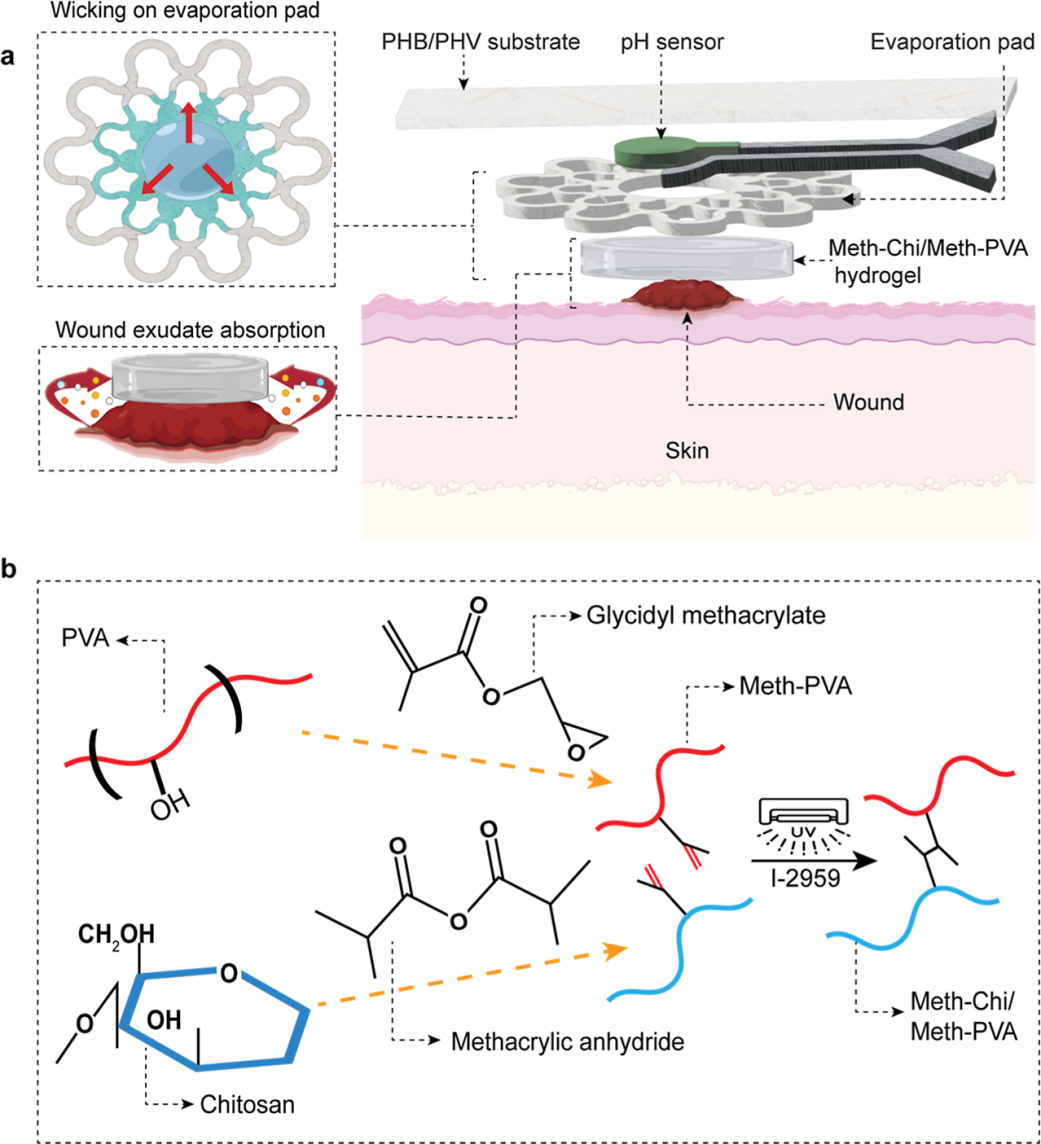
Design of the hydrogel-integrated wound patch. (a) Detailed illustration of the layer-by-layer layout of the patch with a schematic representation of its working principle. (b) Preparation process of the Meth-Chi/Meth-PVA hydrogel.

### Characterization of Meth-Chi/Meth-PVA

Meth-Chi/Meth-PVA hydrogels were obtained by UV cross-linking after evenly mixing Meth-Chi and Meth-PVA to create an antibacterial and porous contact area between the sensor and skin (Figure 2a). The porosity of the synthesized hydrogels was verified by SEM visualization. Figure 2b illustrates that Meth-Chi/Meth-PVA hydrogels are highly porous, featuring pore sizes approximately between 15 and 20 μm. Figure 2c shows the FTIR spectra of chitosan and Meth-Chi. Common peaks at 2850 and 2935 cm^–1^ indicate the alkyl C–H stretching, a group present in both.^35^ The appearance of a new peak at 1657 cm^–1^ refers to the new amide C=O stretch,^36^ the peak at 1670 cm^–1^ is attributed to C=C stretching vibration of the vinyl group, the peak at 880 is due to the C–H wagging stretching of the vinyl group, and the peak at 1187 is attributed to the C–O stretching vibration of the alcohol group.^37^ The FTIR spectra of PVA and methacrylated PVA are shown in Figure 2d. For Meth-PVA spectra, the peaks at 1725 and 1637 cm^–1^ correspond to the stretching vibrations of the C=O and C=C bonds, respectively.^36^ Additionally, a peak at 1023 cm^–1^ indicates the stretching vibrations of the C–O (alcohol) groups, observed in both PVA and Meth-PVA spectra but with greater intensity in Meth-PVA. The peak at 944 cm^–1^ is identified as the out-of-plane bending of R_2_C–CH_2_ of Meth-PVA.^38^ Altogether, the successful methacrylation of chitosan and PVA was confirmed by an FTIR analysis.

**Figure 2:**
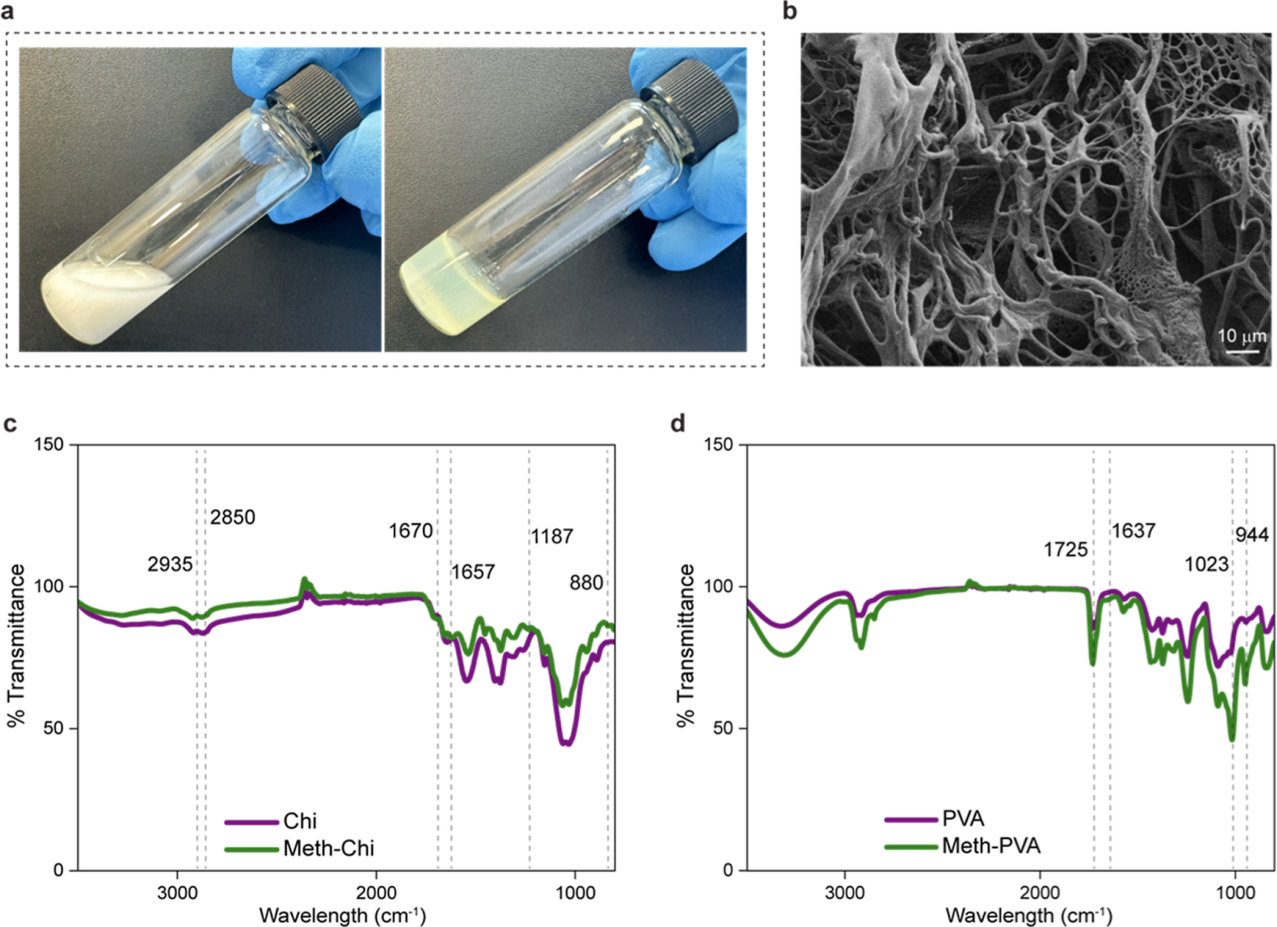
Meth-Chi/Meth PVA hydrogel characterization. (a) Photographs of Meth-Chi/Meth-PVA before (left) and after (right) photopolymerization. (b) SEM image of the Meth-Chi/Meth PVA hydrogel. FTIR spectra of (c) Chi and Meth-Chi and (d) PVA and Meth-PVA.

### Characterization of the Sensing System

The sensing part of the biodegradable patch is in continuous contact with wound exudate to give us enough information regarding wound pH. In order to guarantee precise and reliable pH detection, sensing performance metrics of the pH sensor, including its sensitivity, selectivity, stability, and reproducibility, were carefully investigated. The pH detection was accomplished by a two-electrode method with an ion-selective electrode and a Ag/AgCl reference electrode. Initially, in vitro potentiometric measurements with benchtop equipment (CHI660E) were conducted with artificial wound exudate (AWE) solutions with pH values in the range of 5.153–8.22, which is relevant to the physiological pH values of wound exudate.^39^ Briefly, to realize the pH sensor, the carbon working electrode was coated with an electropolymerized polyaniline (PANi) layer, which acts as the hydrogen selective layer (Figures 3a and S1, Supporting Information).

**Figure 3:**
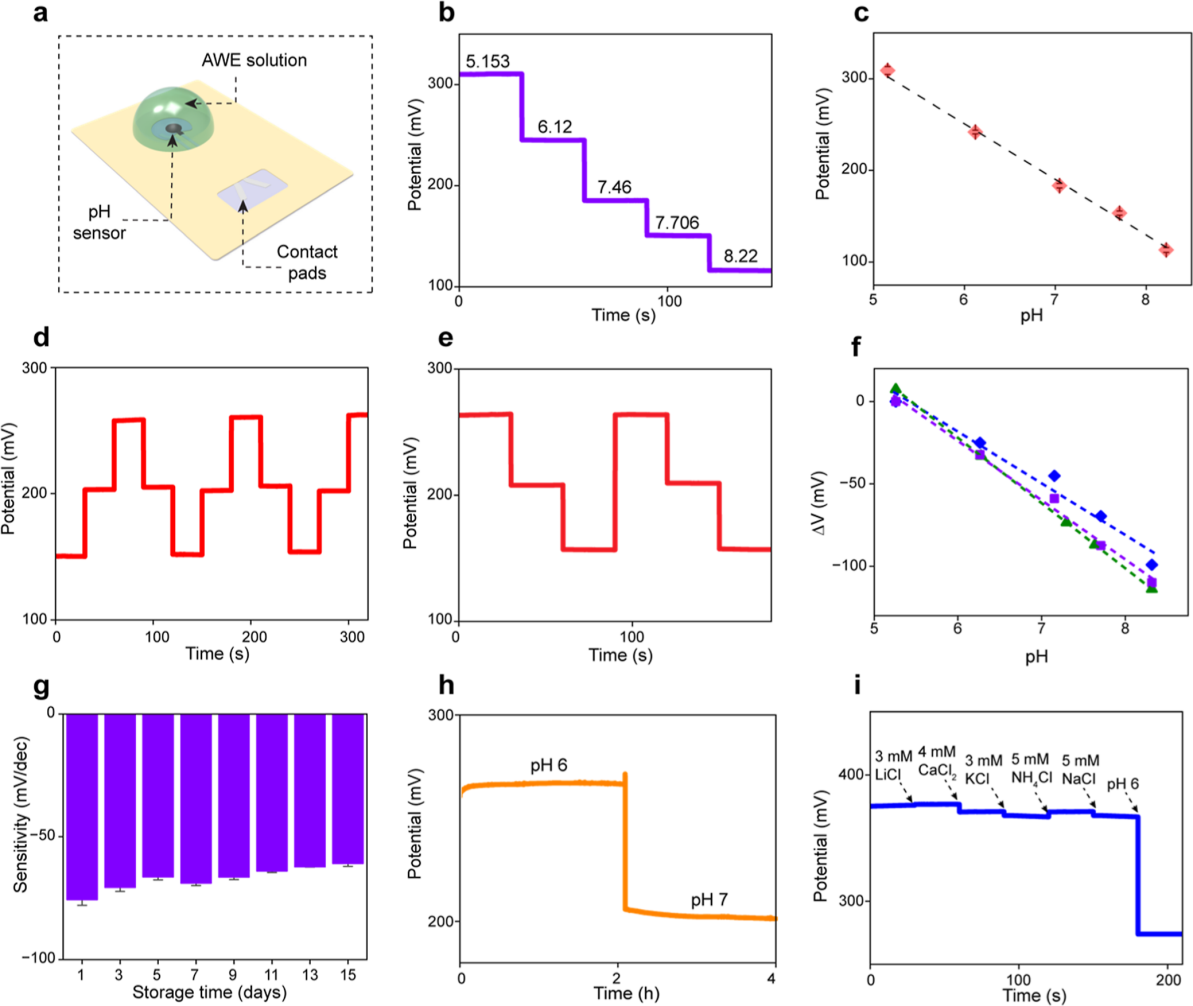
Experimental characterization of the pH sensor. (a) Schematic representation of the pH sensing setup. (b) OCP response of the pH sensor measured for different pH values. (c) The corresponding calibration curve (*n* = 5). (d) Reversibility and (e) recovery test at pH levels of pH_1_ = 6.12, pH_2_ = 7.046, and pH_3_ = 7.706 in AWE. (f) Reproducibility of the pH sensors (*n* = 3). (g) Long-term stability of the pH sensor after 15 days of storage. (h) Response stability of the pH sensor in two different pH solutions. (i) Selectivity study of the pH sensor against other possible interferents.

The sensitivity of the pH sensor was assessed by measuring the change in the open circuit potential (OCP) response of the pH sensor upon additions of AWE solutions with different pH values. The measured potentiometric signals revealed the concentration-dependent stepwise downward trend of the OCP values of the pH sensor in response to increasing values of AWE pH (Figure 3b). As can be seen in Figure 3c, the pH sensor demonstrated a linear voltage response of 62.29 mV per pH unit, indicative of near-Nernstian behavior, with a correlation coefficient and maximum relative standard deviation (RSD) of 0.998 and 2.38%, respectively.

The reversibility of the potentiometric sensors is essential for reliable measurements of target analytes over long periods. The carryover analysis was performed by exposing the pH sensor to AWE solutions with various pH values in a cyclic and staircase manner. The sensors were tested by exposing them to pH levels ranging from 6.94 to 9.23 in incremental steps to assess the impact of sudden changes in pH on sensor performance. As can be seen in Figure 3d, the abrupt and large variations in pH values had a negligible influence on the continuous sensor performance with a minimal deviation of 0.93%. The response signal stabilized within 30 s and remained consistent during the measurements. Previous studies have suggested faster response times due to variations in the PANi layer thickness. However, for wound monitoring applications, immediate response times are less critical because pH levels do not fluctuate significantly in a short time frame.^23^ To examine the recovery behavior of the pH sensor, the sensors were exposed to various pH values in a staircase manner. As can be seen in Figure 3e, the abrupt and large variations in pH values caused minor changes in the continuous sensor performance with a minimal deviation of 4.13%. The reproducibility of the pH sensor was explored by measuring the potentials of five sensors in response to the same pH ranges. As can be seen in Figure 3f, the sensor exhibited good reproducibility over a wide pH range, with a deviation (RSD) of 9.04%.

As the duration of wound healing among individuals generally takes 2–3 weeks,^40^ thereby suggesting the importance of long-term stability of the sensors for continuous monitoring applications, we recorded the potentiometric response of the sensors every other day for 2 weeks. Figure 3g demonstrates that the sensor sensitivity remained stable, with an average deviation of 13%, indicating the pH sensor’s reliability over the average wound healing duration. In a separate experiment, a 4 h measurement was conducted to demonstrate the sensor’s stability for continuous monitoring in two different pH levels. As shown in Figure 3h, the RSD values were 0.29% and 0.58% for pH 6 and pH 7, respectively, depicting sufficient stability of the sensor for long-term pH detection. Since wound exudate fluid contains diverse ions and potential interferences, the sensor’s selectivity is critical for wound monitoring applications. As evidenced in Figure 3i, the results revealed that the maximum interference effect was induced by the addition of KCl, with a corresponding potential change of approximately 1.94%. This confirms the sensor’s high selectivity, indicating its great potential for continuous pH monitoring in wound environments.

To further demonstrate the feasibility of our sensor beyond a bulky benchtop setup, we integrated it into a custom-developed board (Figure S2) and evaluated its performance using three key measurements, as demonstrated in Figure S3. A clear linear relationship (Figure S3a) between the measured potential and pH is observed across acidic, neutral, and alkaline solutions (pH 4–10), with 25.63 mV per pH unit indicative of accurate sensor calibration. In Figure S3b, we highlight the reversibility of the sensor’s response by sequentially switching between three pH levels; the stable, repeatable potential shifts confirm that the sensor returns to baseline each time. Lastly, in Figure S3c, the sensor’s rapid recovery when transitioning from high to low pH (and vice versa) demonstrates robust real-time performance. Overall, these results validate both the sensor’s consistent pH sensitivity and its reliable operation in a more portable, board-based configuration, underscoring its potential for wearable wound-monitoring applications.

Finally, the mechanical properties of the employed substrate were examined in order to assess the mechanical behavior of the developed patch under mechanical deformations. The developed wound dressing comprises a double-layered structure consisting of a PHB/PHV substrate and a Meth-PVA/Meth-Chi hydrogel layer. Accordingly, the mechanical strength was evaluated through tensile testing of the PHB/PHV substrate in three trials. The results revealed an average ultimate tensile strength (UTS) of 17.57 MPa, well within the ideal range, and a remarkably low RSD of 0.52%, highlighting the substrate’s consistent and dependable mechanical properties (Figure S4, Supporting Information).^41^ We further characterized the sensing performance of the pH sensor upon undergoing various mechanical deformations such as bending and twisting (Figure S4, Supporting Information).

### Meth-Chi/Meth-PVA Hydrogel Integration with the pH Sensor

The Meth-Chi/Meth-PVA hydrogel is integrated into the patch to collect wound exudate and improve the contact between the sensor and skin (Figure 4a). Wound exudate absorption leads to the swelling of the hydrogel, which acts as a medium to initiate electrochemical reactions on the sensor. The sensing starts upon contact of the absorbed liquid with the electrodes. We exploited this characteristic of hydrogel to circumvent the need to perform extra tasks such as hydrogel centrifugation for extraction of the target biomarker, thereby enabling a more practical tool for continuous wound monitoring.^42^ The integration of the hydrogel layer ensures continuous pH monitoring of the wound region as it retains water in its matrix for an extended period. To illustrate this, we examined the sensing performance of the sensor by placing the as-prepared wet hydrogel layer and wet cellulose paper on two separate pH sensors, followed by measuring the OCP response over a long period. The Meth-Chi/Meth-PVA hydrogel and cellulose paper were soaked in a pH 5 solution until they were completely wet. Upon placing them on the pH sensors, the potentiometric response of the pH sensor was recorded for 130 min. The paper-integrated sensor showed unstable responses after about 80 min, while the OCP response of the hydrogel-integrated sensor remained stable for the whole period (Figure 4b). Hydrogels’ water-retaining capacity not only benefits continuous biomarker monitoring but also improves the wound healing process by keeping the wound area moist.^40^ Among different hydrogels, chitosan is highly prominent due to its antibacterial properties, biocompatibility, and biodegradability.^40,41,43^ Additionally, we combined PVA with chitosan to enhance the hydrogel’s adhesive properties, improving sensor attachment and measurement accuracy. In the literature, chitosan/PVA hydrogels are frequently prepared using cross-linking agents such as glutaraldehyde,^44^ formaldehyde,^45^ and boric acid,^46^ which might lead to further damage to the wounded human skin or tissue because of their toxic properties.^47^ To overcome this issue, we utilized nontoxic I-2959 as the photoinitiator.^48^

**Figure 4:**
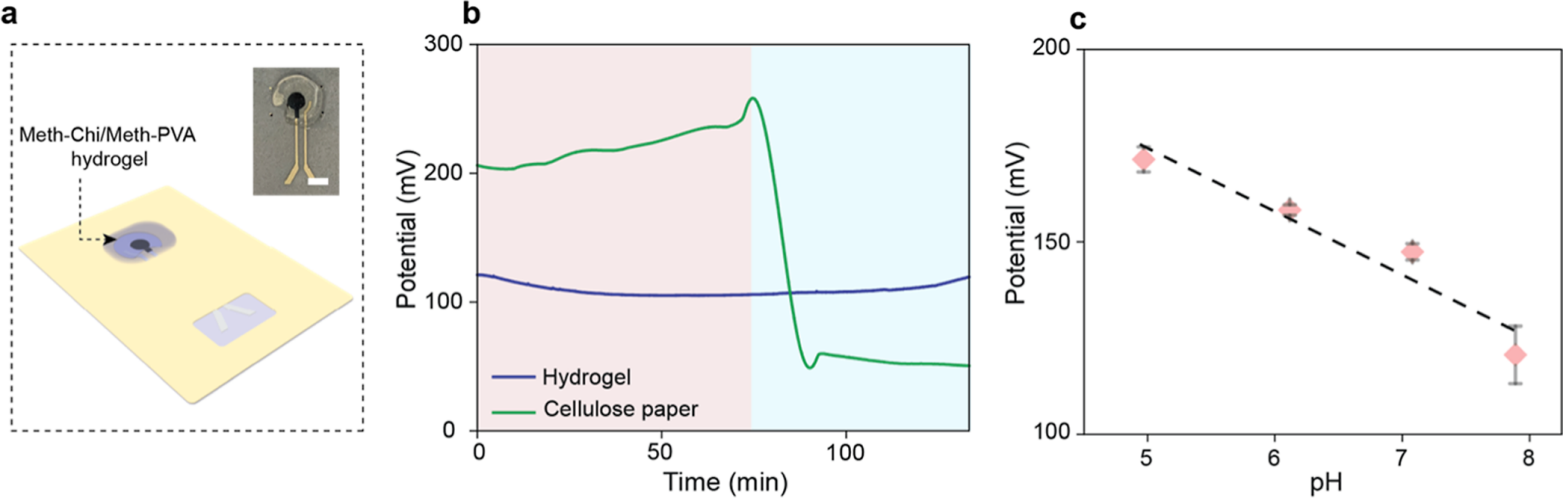
Hydrogel integration with the pH sensor for electrochemical analysis. (a) Schematic representation and optical image (inset) of hydrogel integration into the pH sensor. (b) Stability response of the hydrogel-integrated pH sensor compared to cellulose paper. (c) Calibration curve plot of the hydrogel-integrated pH sensor (*n* = 3).

In order to verify that the integration of hydrogels with the system does not result in an adverse effect on pH sensitivity, we further characterized the sensing performance of the hydrogel-integrated pH sensor. The hydrogel samples of the same size (∼80 mm^2^) were submerged in AWE solutions with pH values of 5, 6, 7, and 8 for 3 h to ensure an equilibrated state, followed by immediate placement of the hydrogel samples on the pH sensors. As shown in Figure 4c, the pH sensor exhibited a linear response of 16.92 mV per pH, with a correlation coefficient and maximum RSD value of 0.96 and 6.2%, respectively. Overall, the in vitro electrochemical sensing results revealed satisfactory performance of the hydrogel-integrated pH sensor in continuous wound monitoring.

Most wound dressing products are single-use, necessitating the development of materials that are not only effective in wound care but also environmentally friendly. We utilized biodegradable and biocompatible materials for the development of different parts of the wound monitoring patch, rendering it an eco- and user-friendly device. To confirm that the developed patch can fully biodegrade naturally in the soil, we conducted a soil degradation study over a period of 30 days for the substrate. The PHB/PHV film was buried in the soil under controlled environmental conditions. Regular observations were made at determined intervals to monitor the progression of biodegradation. On the 15th day, initial signs of degradation were observed as small holes began to appear on the surface of the sensor substrate. The presence of these small holes suggested that the sensor material is being effectively broken down by the microorganisms. By the end of the 30 day period, the entire substrate of the sensor was fully degraded, leaving behind only the silver paste area used in the sensor’s construction (Figure S5). The complete degradation of the sensor’s components underscores its biodegradability, rendering it an eco-friendly option for disposable wound dressing applications.

### Integration of the Meth-Chi/Meth-PVA Hydrogel with Cellulose Paper as an Evaporation Pad

The long-term absorption of wound exudate can result in the saturation of the Meth-Chi/Meth-PVA hydrogel, hindering its ability to absorb fresh wound exudate and thereby complicating the efficacy of monitoring the healing process. The integration of paper, an inexpensive and readily available material, could act as a driving force to extract the absorbed exudate out of the hydrogel through capillary wicking (Figure 5a). As the fluid wicks through the paper, it will evaporate at the evaporation area, which is in contact with the air (Figure 5b). First, the swelling ability of the Meth-Chi/Meth-PVA hydrogel was investigated by placing the hydrogel samples in AWE solutions and measuring the mass change over time. As shown in Figure 5c, the swelling ratio of the hydrogel samples reached a plateau within about 30 min when the mass of the hydrogel samples was increased by about 350%. This swelling ratio represents the expected lifespan of Meth-Chi/Meth-PVA for continuous usage of the developed hydrogel in wound monitoring. The evaporation-assisted transport of liquid through a paper-based structure via capillary wicking forms a continuous flow from the hydrogel toward the paper, enabling the refreshing of wound exudate on the sensing electrodes.

**Figure 5:**
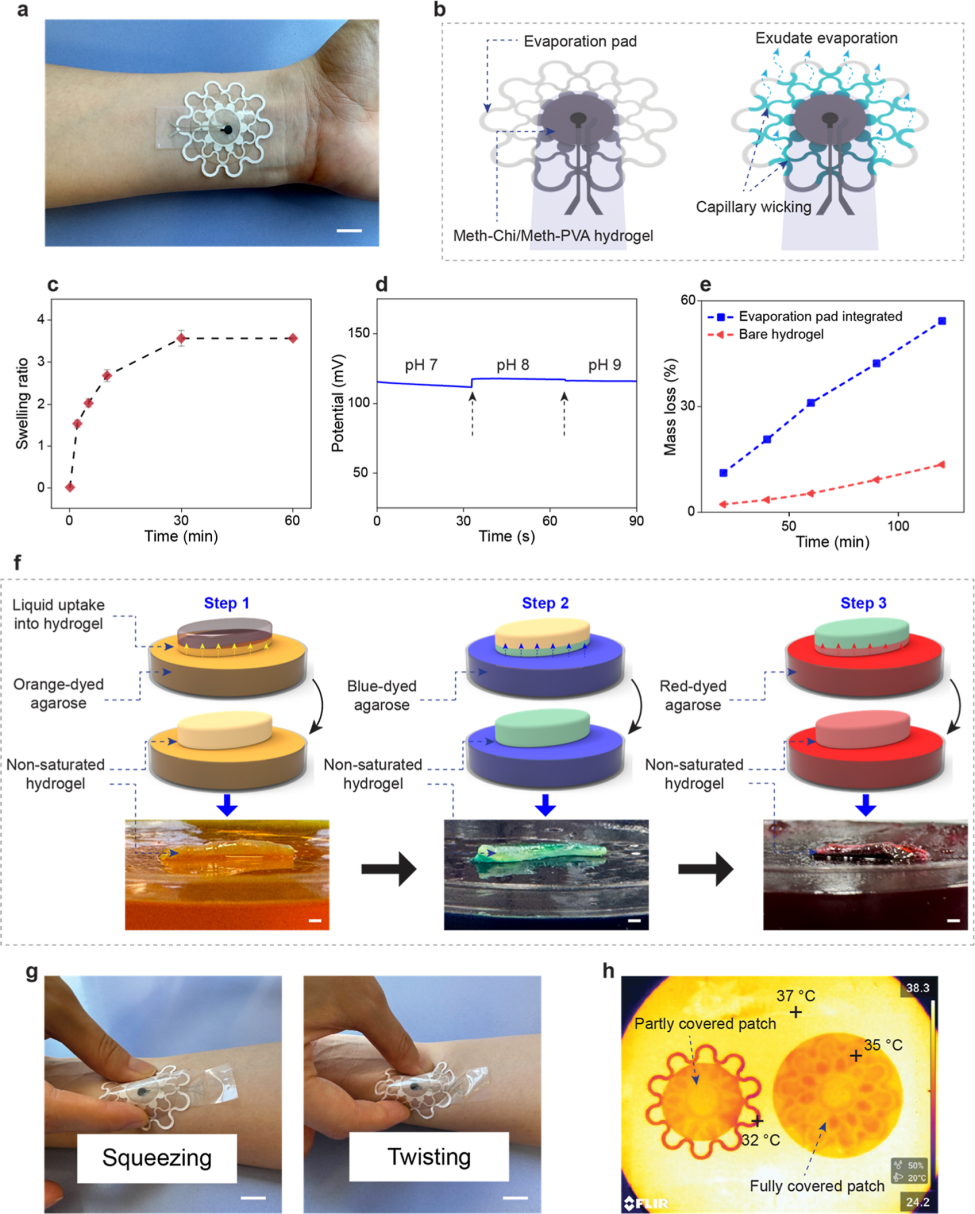
Performance characterization of the capillary-driven wound patch. (a) Optical image of the wound patch worn on a human arm. (b) Working principle of liquid uptake and evaporation through the patch. (c) Swelling behavior of the cross-linked Meth-Chi/Meth-PVA hydrogel. (d) Potentiometric response of the fully saturated hydrogel with an AWE of pH 7, followed by immersing the same hydrogel in AWE solutions of pH 8 and pH 9, in a sequential manner. (e) Effect of integrating an evaporation pad on liquid uptake from the hydrogel. (f) Demonstration of successive liquid uptake from agarose to the nonsaturated hydrogel. (g) On-body photographs of the patch upon mechanical stress. (h) Infrared images showing the temperature drop on the exposed regions of the evaporation pad. Scale bars: 1 cm in (a,g) and 1 mm in (f).

To investigate the adverse impact of hydrogel saturation on long-term electrochemical sensing, a hydrogel sample was fully saturated in an AWE solution of pH 7, followed by measuring the OCP response of the hydrogel-integrated pH sensor. Next, the saturated hydrogel sample was immersed in AWE solutions of pH 8 and 9, sequentially, and their potentiometric measurements were performed upon integration into the pH sensor. As can be seen in Figure 5d, the OCP response of the hydrogel-integrated pH sensor exhibited minimal changes of 3.6% and 2.2% for pH values of 8 and 9, respectively. This underscores the necessity for a system capable of continuous liquid uptake from the hydrogel to enable real-time and continuous pH measurement. Given that the evaporation rate from the hydrogel alone may not be adequate for efficient exudate refreshing, we incorporated another layer composed of filter paper into the patch. The paper-based layer is intended to enhance the liquid absorption capacity, thereby complementing the hydrogel’s evaporation rate and ensuring more effective exudate refreshment. As exhibited in Figure 5e, the integration of filter papers into hydrogel samples (10 × 10 mm^2^) resulted in an average increase of 506.11 ± 71.74% in mass loss (evaporation rate). In addition, we investigated the flow rate of the liquid through the evaporation pad to compare it with the flow rate of a moderately exuding wound, which is approximately 10 μL/min. As shown in Figure S6, the measured flow rate was 155.88 μL/min. The flow rate of the liquid through the evaporation pad is sufficient to ensure continuous circulation between the wound, hydrogel, and evaporation pad.

We further demonstrated that the prepared hydrogels are capable of sustained liquid uptake when not fully saturated by employing colorimetric techniques. First, the hydrogel sample was partly saturated by placing it on an orange-dyed agarose gel (Figure 5f, step 1). In step 2, despite the hydrogel sample being entirely orange from the previous step, it was still capable of further blue-dyed liquid uptake (Figure 5f, step 2). The hydrogel sample showed similar behavior upon placing it on the red-dyed agarose gel (Figure 5f, step 3). In addition, a potentiometric measurement was performed to verify the effectiveness of integrating the evaporation pad into the device in exudate renewal during continuous liquid uptake. First, an agarose hydrogel was prepared at pH 7.0, followed by integrating the Meth-Chi/Meth-PVA hydrogel, screen-printed sensor, and evaporation pad. After the potential response of the pH sensor was recorded, a new solution with a pH value of 8.0 was added to the agarose. Upon absorption of the old solution (pH 7.0) by both the hydrogel and the evaporation pad, the new solution (pH 8.0) was able to fully saturate the agarose, thereby ensuring continuous renewal of the solution in contact with the pH sensor. The potentiometric results exhibited a distinct, incremental shift in potential as illustrated in Figure S7—approximately 30 mV—confirming successful renewal of the liquid content within the wound-mimicking medium (agarose hydrogel), rendering it an acceptable candidate for continuous monitoring of wound pH. In summary, these findings confirm the hydrogel’s ability for continuous liquid absorption, provided it remains below saturation. However, as discussed before, once the hydrogel reaches full saturation, its capacity to absorb further liquid is inhibited (Figure S8, Supporting Information). Therefore, an extra system is necessary to ensure continuous exudate uptake from the hydrogel. The effect of evaporation on liquid uptake was further investigated by placing two fully saturated filter papers by a red-dyed liquid on a blue-dyed agarose. One of the filter papers was fully covered with a thin PDMS sheet to minimize the evaporation rate. As evidenced by the RGB values provided in Figure S9 (Supporting Information), the lower evaporation rate of the covered filter paper led to the reduced blue-dyed liquid uptake.

The evaporation pad was designed in a serpentine layout to provide more flexibility and stretchability while ensuring the minimum surface area needed for evaporation (Figure 5g). Additionally, the cooling effect of evaporation was illustrated to show the liquid evaporation on the pad. Two serpentine evaporation pads were placed on a hot plate with a temperature of 37 °C. One evaporation pad was fully covered with PDMS to prevent evaporation, while the other one was partly covered to allow evaporation. A thermal camera (FLIR-E96) was used to capture the temperature difference between evaporating and nonevaporating areas. Figure 5h illustrates a temperature drop of about 3 °C in the evaporation areas due to the cooling effect. Overall, the incorporation of a hydrogel and paper-based evaporation pad into wound monitoring patches can offer great promise for continuous measurement of various biomarkers.

## Conclusions

In this work, we developed a hydrogel-integrated wound pH monitoring patch combined with bioresorbable and ecofriendly materials. The hydrogel, synthesized using methacrylated chitosan and methacrylated poly­(vinyl alcohol), demonstrated good swelling properties. The integration of a serpentine-shaped paper-based microfluidic layer enabled enhanced evaporation and long-term exudate refreshing compared to those of other single-use wound monitoring devices. In vitro pH monitoring was stable and accurate, and its substrate biodegraded within a month, proving its environmental sustainability.

However, this study lacks in vivo testing to validate its performance under conditions such as varying wound exudate flow rates and biochemical interferences. Future work could focus on integrating wearable electrochemical sensing units and conducting in vivo experiments to fully assess the system’s applicability. Additionally, the limited lifespan of the cellulose paper-based evaporation pad restricts the patch’s long-term usability. Identifying more durable materials for the evaporation pad could improve robustness and extend the device’s operational lifetime. Studies on evaporation pads may improve their use in various wearable systems such as interstitial fluid (ISF) monitoring devices or tear-based sensors for long-term monitoring. Expanding the patch’s biomarker features beyond pH could enhance wound monitoring. Our patch proves long-term wound pH monitoring with its integrated evaporation pad and hydrogel medium. This combination paves the way for advancements in developing environmentally friendly, biodegradable wearable devices.

## Experimental Section

### Chemicals and Materials

The PHB92/PHV8 biopolymer was purchased from GoodFellow (PA, USA). Aniline was obtained from Alfa Aesar (MA, USA). The conductive silver and carbon pastes were purchased from Dupont (DE, USA). Bovine serum albumin was purchased from Merck Millipore (MA, USA). Sodium chloride (NaCl), sodium bicarbonate (NaHCO_3_), sodium citrate, sodium lactate, calcium chloride dihydrate (% CaCl_2_·2H_2_O), magnesium chloride (MgCl_2_), lithium chloride (LiCl), calcium chloride (CaCl_2_), potassium chloride (KCl), ammonium chloride (NH_4_Cl), 2-hydroxy-4′-(2-hydroxyethoxy)-2-methylpropiophenone (I-2959), methacrylic anhydride (MA), glycidyl methacrylate (GMA), poly­(vinyl alcohol) (PVA), chitosan, iron­(III) chloride (FeCl_3_), and Whatman grade 1 filter paper were purchased from Sigma-Aldrich (MO, USA). Transparent poly­(ethylene terephthalate) (PET) films were purchased from MG Chemicals (Burlington, Canada). Glucose and pure crystalline urea were obtained from neoFroxx (Hesse, Germany). Hydrochloric acid (HCl), dimethyl sulfoxide (DMSO), acetic acid, acetone, and sodium hydroxide (NaOH) were purchased from ISOLAB (Germany). 4-Dimethylaminopyridine (DMAP) was purchased from abcr GmbH (Germany). Polydimethylsiloxane (PDMS) (Sylgard 184 Silicone Elastomer) was purchased from Dow Corning (MI, USA).

### Artificial Wound Exudate (AWE) Formulation and Ex Vivo Test

AWE was prepared according to the described protocol in the literature.^49^ It consists of 2% bovine serum albumin, 0.36% NaCl, 0.05% NaHCO_3_, 0.02% sodium citrate, 0.1% sodium lactate, 0.1% glucose, 0.01% CaCl_2_·2H_2_O, 0.02% MgCl_2_, and 0.01% urea. The pH of AWE solutions was adjusted using 1 mol/L HCl and 1 mol/L NaOH within a pH range of 5.153–8.22 using a commercial pH meter (Orion Dual Star, Thermo Scientific, MA, USA).

### Sensor Preparation

Electrochemical sensors, comprising carbon working electrodes against Ag/AgCl reference electrodes, were fabricated through screen-printing techniques on a commercial PHB/PHV substrate owing to its biodegradable properties.^50^ The stencil patterns were designed using AutoCAD (AutoDesk, San Rafael, CA, USA) and transferred onto 40 μm PET films using a laser-cutting machine. Separate stencils were developed for the silver (Ag) and carbon electrodes. First, the silver ink was screen printed on the PHB/PHV substrate through a Ag stencil to create the reference electrode and contact pads. Then, the working electrode was patterned on the substrate by using the carbon conductive paste. After each layer deposition, the pastes were cured at 70 °C for 30 min. The Ag/AgCl electrodes were realized by dropping a 0.1 M solution of FeCl_3_ for 1 min on the surface of Ag electrodes. After screen printing, a 500 nm insulation layer of parylene-C was deposited using the SCS Labcoater Parylene Deposition system (IN, USA). Following sensor preparation, electropolymerization of PANi was performed in a solution of 0.1 M aniline in 1 M HCl by cyclic voltammetry from −0.2 to 1 V for 15 cycles (versus the Ag/AgCl electrode) at a scan rate of 50 mV/s. The PANi-coated sensors were washed and dried with N_2_. The schematic representation of the fabrication steps can be found in Figure S1 (Supporting Information).

### Hydrogel Synthesis

Methacrylated chitosan (Meth-Chi) was synthesized using methacrylic anhydride (MA) with minor adaptations to a procedure described in the literature.^35^ In summary, 1.5% w/v chitosan flakes (MW 50 000–190 000 Da) were dissolved by vigorous stirring in 1% v/v acetic acid at room temperature for 6 h. MA (0.6 v/v %) was slowly added to the chitosan solution, and the mixture was stirred at 100 rpm for 12 h at a temperature of 40 °C while being shielded from light. The resultant products were redispersed in deionized water and purified by dialysis (molecular weight cutoff (MWCO) 12–14 kDa membrane) against 4.5 L of deionized water, undergoing six water changes over 72 h. The methacrylation of PVA was conducted using a procedure previously described in the literature.^51^ Initially, a 10% w/v solution of PVA was prepared in 10 mL of DMSO. DMAP and GMA were then added to the solution at concentrations of 1.0 and 0.025 mol %, respectively. This mixture was stirred continuously for 6 h at a temperature of 60 °C. Following the reaction, the mixture was precipitated with acetone and dried under vacuum for 48 h.

Meth-Chi and Meth-PVA solutions were mixed in a 1:1 ratio. The polymer solution was mixed with a 0.5% v/w photoinitiator (I-2959) and stirred at 300 rpm for 10 min, resulting in a homogeneous polymer solution. Subsequently, the prepared solution was exposed to 320 nm UV light with an intensity of 175 mW/cm^2^ for 20 min (Uvitron INTELLI-RAY 600). Samples were rinsed with distilled water 2–3 times to remove unreacted residuals and subsequently dried at 37 °C for 4 h to ensure the removal of uncross-linked polymer solution.

### In Vitro Electrochemical Detection of pH

Electrochemical measurements between the PANi-coated and reference electrodes were conducted by a standard potentiometric technique via a CHI660E electrochemical workstation (Shanghai Chenghua Instrument Co. Ltd., Shanghai, China). The characterization of sensors was evaluated by adjusting the pH levels by using NaOH (0.1 M) and HCl (0.1 M) solutions. To calibrate the sensor, 100 μL of AWE with different pH levels was dropped on the electrode array, and potentiometric measurement was conducted. Afterward, the solution was removed, the surface was gently cleaned of any remaining residue, and then the next solution was applied. A commercial pH meter (Orion Dual Star, Thermo Scientific, MA, USA) was used to measure the solutions’ pH and compare them with the detected pH values. The sensitivity experiment was performed by successive addition of solutions with pH values of 5.153 and 8.22 on the electrode and recording the OCP for 60 s with a sample frequency of 10 Hz. Upon changing the solutions, the measurement was temporarily paused. In order to study the selectivity performance of the pH sensor, physiologically relevant concentrations of LiCl, CaCl_2_, KCl, NH_4_Cl, and NaCl solutions were added to the AWE solution as interferents. The stability of the pH sensor was examined in two different experiments. The initial experiment involved conducting OCP measurements for a duration of 4 h, with the sensor exposed to solutions of pH 6 for 2 h, followed by pH 7 for another 2 h. In a separate experiment, the OCP response of the electrode was monitored over a period of 2 weeks to ensure sensor consistency.

### In Vitro Electrochemical Detection of pH with a Custom-Developed Board

Electrochemical measurements between the PANi-coated and reference electrodes were conducted by a standard potentiometric technique via a custom-developed board. The characterization of sensors was evaluated by adjusting the pH levels using NaOH (0.1 M) and HCl (0.1 M) solutions. To calibrate the sensor, 100 μL of AWE with different pH levels (pH_1_ = 4, pH_2_ = 7, and pH_3_ = 10 in AWE) was dropped on the electrode array, and potentiometric measurement was conducted. Afterward, the solution was removed, the surface was gently cleaned of any remaining residue, and then the next solution was applied. A commercial pH meter (Orion Dual Star, Thermo Scientific, MA, USA) was used to measure the solutions’ pH and compare them with the detected pH values.

### In Vitro Electrochemical Detection of pH with Hydrogel-Integrated Sensors

Hydrogel pieces (2 × 2 cm^2^) were prepared and immersed in AWE solutions with pH levels of 5, 6, 7, and 8 for 60 min to allow complete saturation of the hydrogel pieces. Upon removal of the pieces from the solutions, cellulose paper was used to absorb the liquid residues, followed by drying using an air gun to eliminate any remaining liquid from the hydrogel surface. The hydrogels containing solutions of different pH values were successively placed on the pH sensor, and the OCP response between the PANi-coated electrode and reference electrode was recorded using a standard potentiometric technique with a CHI660E electrochemical workstation.

### Flow Rate Calculation through an Evaporation Pad

The initial weight of the evaporation pad was measured before the experiment. Hydrogel pieces (*R* = 8 cm) were immersed in green-colored liquid for 3 h to achieve full saturation. The fully saturated hydrogel piece is placed on the preweighed evaporation pad. The hydrogel piece remained on the evaporation pad until the evaporation pad was fully saturated with its color. After the saturation of a pad, the current weight of the evaporation pad was recorded. The absorbed volume of liquid was calculated by dividing the weight difference (measured weight minus initial weight) by the density of the respective liquid. Finally, the flow rate through the evaporation pad was determined by dividing the calculated liquid volume by the time required for saturation.

### Mechanical Tests of PHB/PHV

The tensile test of the PHB/PHV substrate was conducted according to the ASTM D 882-18 standard test method^52^ using an Instron E300 testing machine (Norwood, MA, USA). Samples were cut into (15 × 170) mm^2^ strips using a laser machine. The substrate was placed between jaws set 10 mm apart, and the tensile test was conducted at a traction speed of 5 mm/min until break. Three repetitions were performed, and each measurement was recorded by Bluehill software (Instron, Norwood, MA, USA) as displacement versus load and then converted to a stress–strain curve.

### Swelling Measurements of the Meth-PVA/Meth-Chi Hydrogel

The swelling ability of the as-prepared hydrogels was derived by comparing their masses before and after fluid absorption. The swelling ratio was calculated according to the equation (*M* – *M*_0_)/*M*_0_, in which *M*_0_ and *M* represent the weights of hydrogel samples in the dry and wet states, respectively. First, the dry mass of each sample (*M*_0_) was measured before any treatment. Then, the samples were immersed in AWE solution for 60 min. At each determined time point, the surface water was removed using a cellulose paper and air gun, followed by immediate measurement of the sample mass (*M*). The amount of the extracted absorbed solution by the hydrogel samples can be calculated by *M* – *M*_0_.

### Degradation of the Hydrogel-Integrated pH Sensor

The degradation of the hydrogel-integrated pH sensor was conducted following the PHB/PHV substrate degradation protocol described in the literature.^50^ Approximately 5 cm of soil was laid in a glass container, and then the sensor was placed on top. Another layer of soil was added to ensure a consistent contact with the degradative environment. To maintain a 100% relative humidity, the soil was saturated with water. An additional 25 mL of water was added every 2 days to keep the soil moist. The degradation process was documented by taking photographs on days 1, 15, and 30.

### Design of PCB

The PCB was designed with Altium Designer software (Altium, Australia). The rigid FR4 board was patterned by an ultraviolet (UV) 355 nm laser machine (ProtoLaser U4, LPKF, Germany). All the passive and active components were mounted on the PCB using soldering paste and a JBC NASE-2C precision soldering station. An ultralow power Bluetooth Low Energy (BLE) system on a chip (SoC) (nRF52832, Nordic Semiconductor) was programmed and used as the main processor of the PCB. A voltage divider was designed to obtain the analog voltage values from the pH sensor and convert them to the digital domain using the built-in 12 bit analog-to-digital converter (ADC) unit of the microcontroller.

## Supplementary Material



## Notes

Fabrication process of screen-printed electrodes, integration of the flexible screen-printed electrode with the custom PCB, experimental characterization of the pH sensor with a custom-developed board, electrochemical sensing performance of the pH sensor upon applying mechanical deformations and stress and strain curves of the PHB/PHV substrate, biodegradation of the PHB/PHV substrate, flow rate calculation of an evaporation pad, demonstration of absorption halt after saturation of the hydrogel, and demonstration of the effect of evaporation on liquid uptake (PDF)
