# Supplementary material for: Methacrylated Chitosan Methacrylated Poly(vinyl alcohol)-Based Hydrogel Patch for Long-Term Electrochemical Wound pH Sensing
Source: ACS Sens. 2025 Apr 30;10(5):3347–57. doi: 10.1021/acssensors.4c02172 (PMC12295560; doi:10.1021/acssensors.4c02172)
Supplement: Supplementary file 1 [file se4c02172_si_001.pdf]

Supporting Information

Methacrylated Chitosan Methacrylated Polyvinyl Alcohol-Based Hydrogel Patch for Long-Term Electrochemical Wound pH Sensing

*Maide Miray Albay, Taher Abbasiasl, Çiğdem Buse Oral, Levent Beker**

Maide Miray Albay, Taher Abbasiasl, Çiğdem Buse Oral, and Levent Beker
Department of Biomedical Sciences and Engineering, Koç University
Rumelifeneri Yolu, Sarıyer, Istanbul 34450, Turkey

Levent Beker
Department of Mechanical Engineering, Koç University
Koç University Research Center for Translational Research (KUTTAM), Koç University
Nanofabrication and Nanocharacterization Center for Scientific and Technological Advanced Research (n2Star), Koç University
Rumelifeneri Yolu, Sarıyer, Istanbul 34450, Turkey
E-mail: lbeker@ku.edu.tr

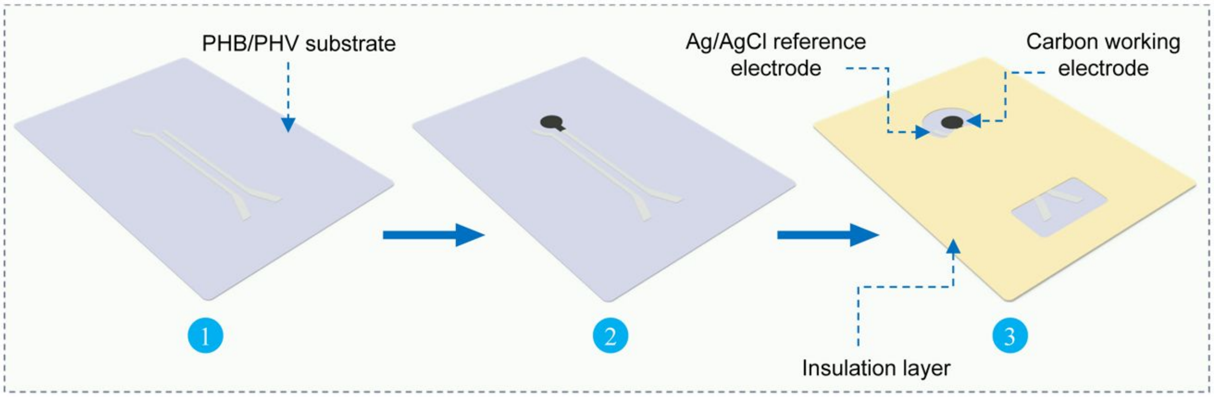

Figure S1. Fabrication process of screen-printed electrodes. (1) Silver layer screen-printed on a PHB/PHV substrate using the first mask for connections and reference electrode. (2) Carbon layer screen-printed using the second mask for working. (3) Parylene-C deposition as the insulation layer.

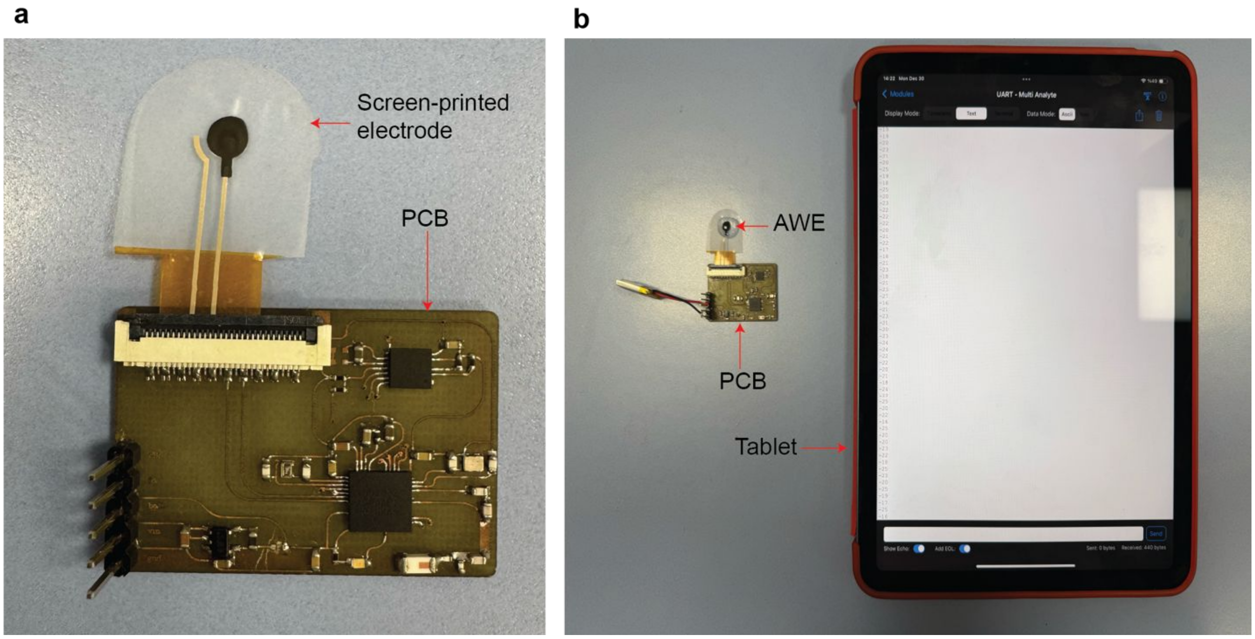

Figure S2. a) Integration of the flexible screen-printed electrode with the custom PCB (Size:30 mmx40 mm). **b)** Complete test setup for pH sensing; the PCB transmits data to a tablet via Bluetooth Low Energy for real-time measurement.

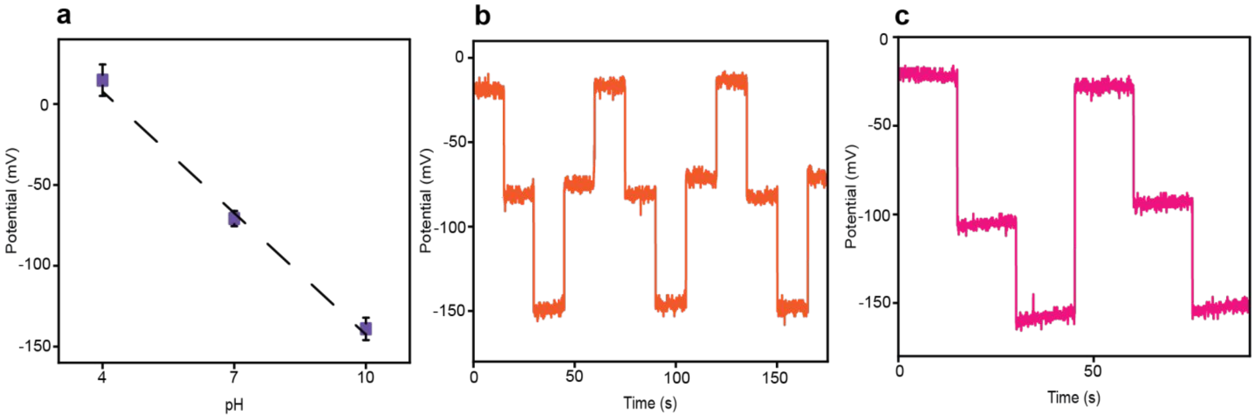

Figure S3. Experimental characterization of the pH sensor with custom developed board. **a)** The corresponding calibration curve (n=5). **b)** Reversibility and **c)** recovery test at pH levels of pH_1 = 4, pH_2 = 7, and pH_3 = 10 in AWE.

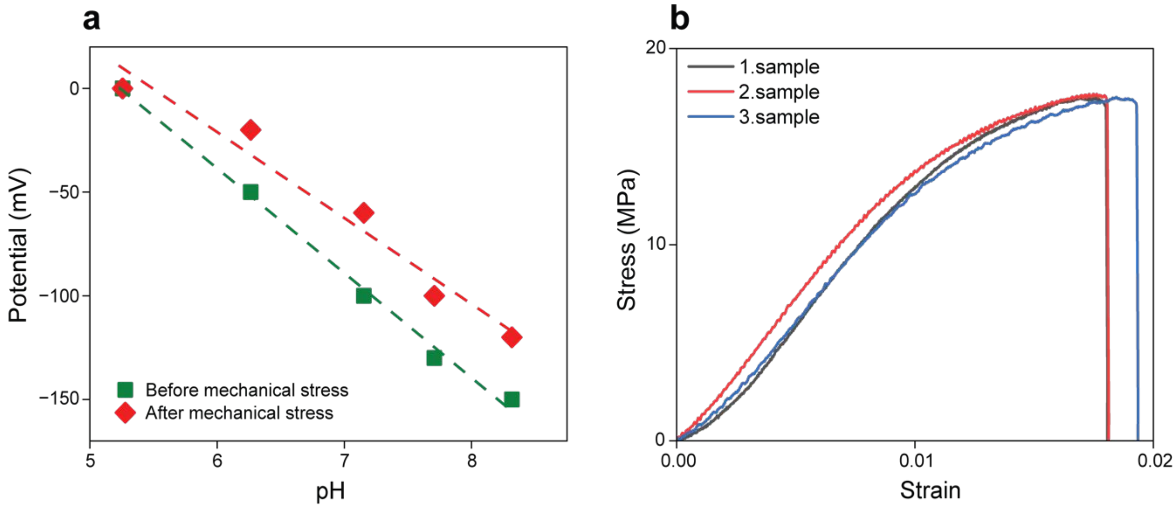

Figure S4. a) Electrochemical sensing performance of the pH sensor upon applying mechanical deformations. **b)** Stress and strain curve of PHB/PHV substrate.

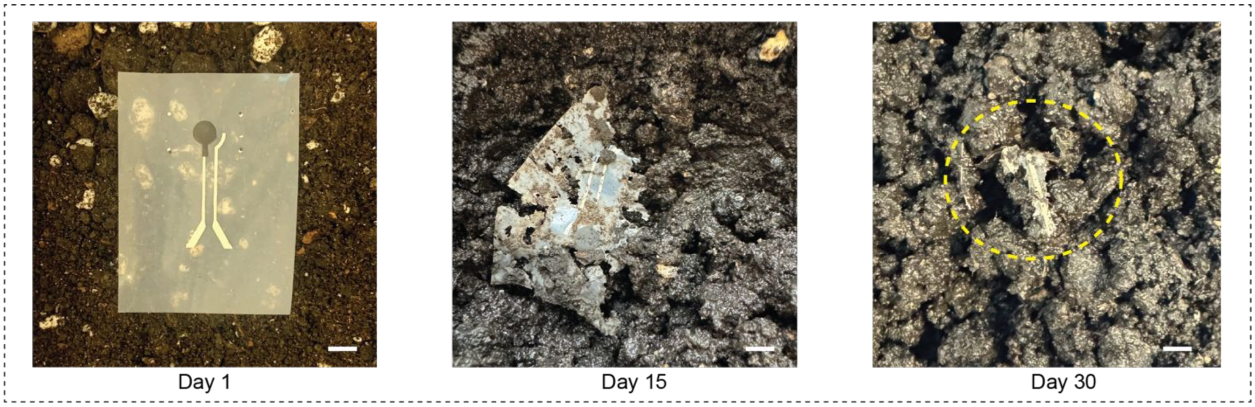

Figure S5. Soil degradation experiment of PHB/PHV substrate. Scale bars are 5 mm.

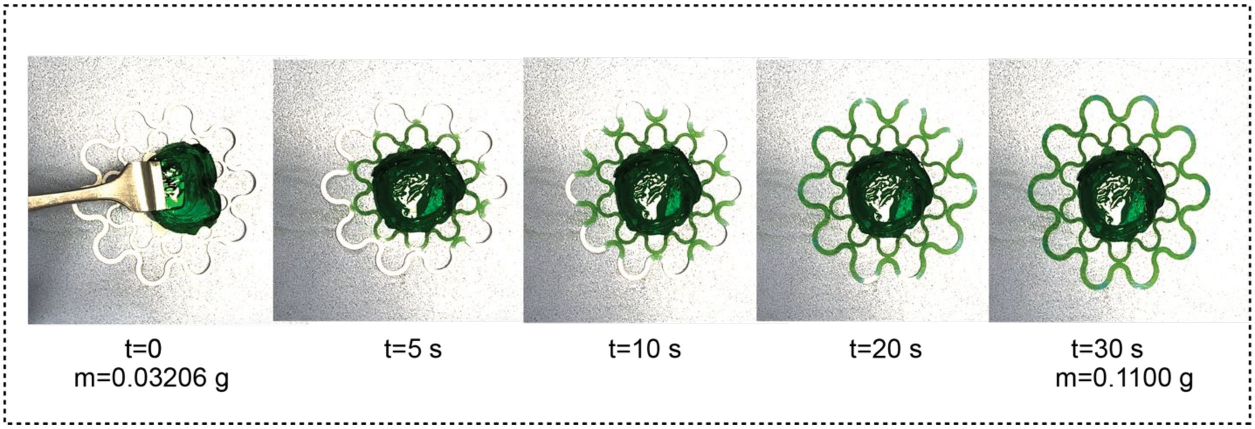

Figure S6. Liquid flow through evaporation pad. Initial weight of the evaporation pad is 0.03206 g, final weight of evaporation pad is 0.01100 g.

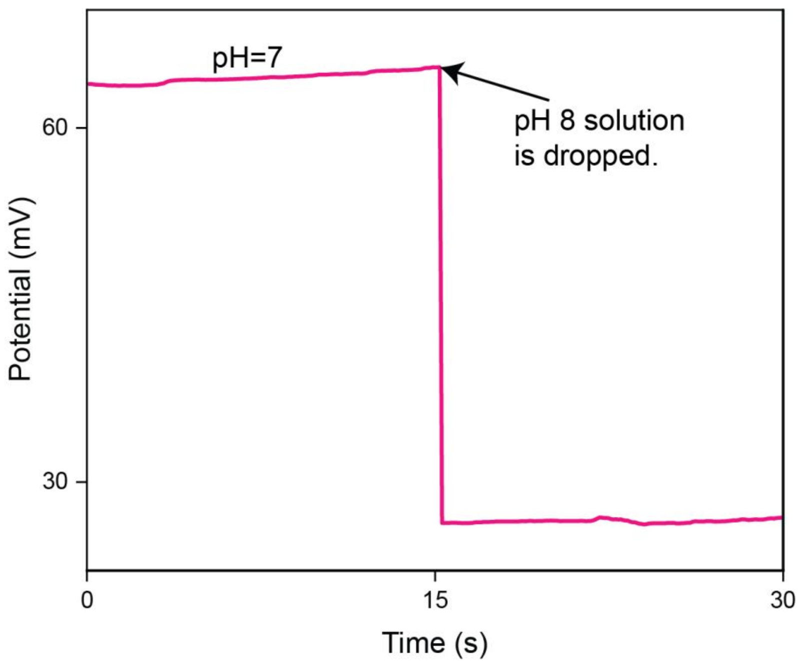

Figure S7. Potentiometric response of the hydrogel-integrated sensor upon changing the pH of the agarose.

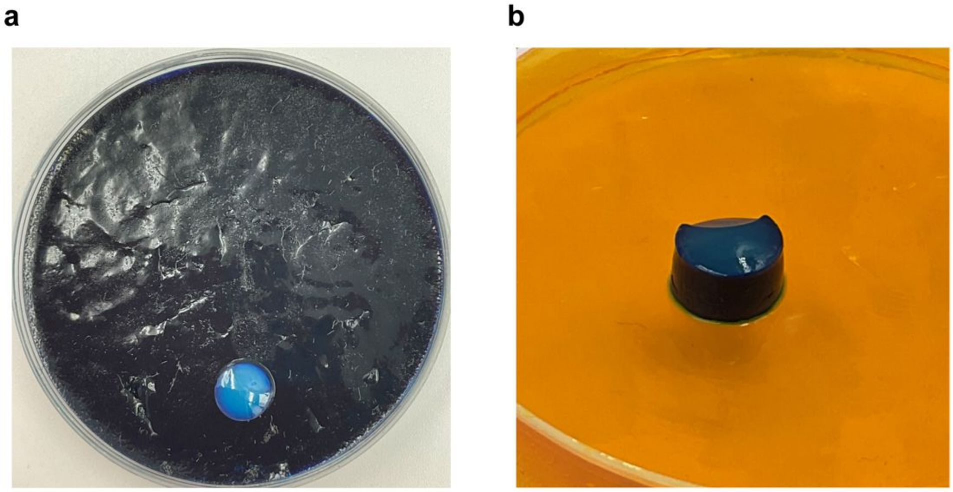

Figure S8. Demonstration of absorption halt after saturation of hydrogel. **a)** First, the hydrogel was saturated by placing it on a blue agarose gel. **b)** No visible color change in the hydrogel after placement on the orange agarose gel.

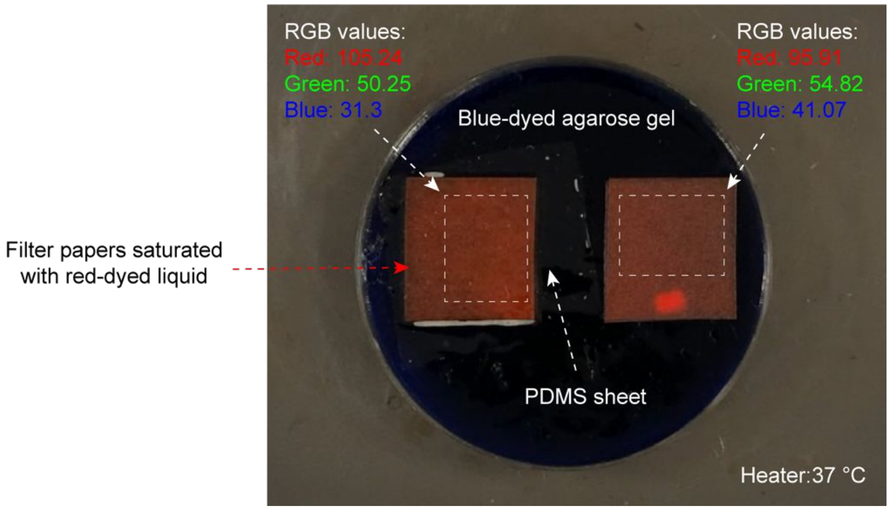

Figure S9. Demonstration of the effect of evaporation on liquid uptake. Filter papers of equal dimensions were saturated with red-dyed liquid, followed by immediate placement on a blue-dyed agarose gel. One of the filter papers (left in the picture) was covered with a PDMS sheet to lower the evaporation rate, while the other (right in the picture) was left exposed to the air. The experiment was performed at 37 ºC. ImageJ was used to extract the R, G, and B values.
